# Chemical Inhibitors of Dynamin Exert Differential Effects in VEGF Signaling

**DOI:** 10.3390/cells10050997

**Published:** 2021-04-23

**Authors:** Dimitris Basagiannis, Sofia Zografou, Evangeli Goula, Despoina Gkeka, Evangelos Kolettas, Savvas Christoforidis

**Affiliations:** 1 Department of Biomedical Research, Foundation for Research and Technology, Institute of Molecular Biology and Biotechnology, 45110 Ioannina, Greece; dimitris.basagiannis@unige.ch (D.B.); szografu@gmail.com (S.Z.); evangeliagoula@gmail.com (E.G.); despinageka@gmail.com (D.G.); ekoletas@uoi.gr (E.K.); 2 Laboratory of Biological Chemistry, Department of Medicine, School of Health Sciences, University of Ioannina, 45110 Ioannina, Greece; 3 Laboratory of General Biology, Department of Medicine, School of Health Sciences, University of Ioannina, 45110 Ioannina, Greece

**Keywords:** cell signaling, endocytosis, vesicular trafficking, dynamin, inhibitors, endothelial cell biology

## Abstract

VEGFR2 is the main receptor and mediator of the vasculogenic and angiogenic activity of VEGF. Activated VEGFR2 internalizes through clathrin-mediated endocytosis and macropinocytosis. As dynamin is a key regulator of the clathrin pathway, chemical inhibitors of dynamin are commonly used to assess the role of the clathrin route in receptor signaling. However, drugs may also exert off-target effects. Here, we compare the effects of three dynamin inhibitors, dynasore, dyngo 4a and dynole, on VEGFR2 internalization and signaling. Although these drugs consistently inhibit clathrin-mediated endocytosis of both transferrin (a typical cargo of this route) and VEGFR2, surprisingly, they exert contradictory effects in receptor signaling. Thus, while dynasore has no effect on phosphorylation of VEGFR2, the other two drugs are strong inhibitors. Furthermore, although dyngo does not interfere with phosphorylation of Akt, dynasore and dynole have a strong inhibitory effect. These inconsistent effects suggest that the above dynamin blockers, besides inhibiting dynamin-dependent endocytosis of VEGFR2, exert additional inhibitory effects on signaling that are independent of endocytosis; i.e., they are due to off-target effects. Using a recently developed protocol, we comparatively validate the specificity of two endocytic inhibitors, dynasore and EIPA. Our findings highlight the importance of assessing whether the effect of an endocytic drug on signaling is specifically due to its interference with endocytosis or due to off-targets.

## 1. Introduction

Endothelial cells generate the inner wall of blood vessels and play a fundamental role in vasculogenesis (de novo blood vessel formation) and angiogenesis (vessel formation from pre-existing vessels) [1], as well as in blood vessel homeostasis [2]. Dysfunction of the endothelial cells is implicated in the most life-threatening diseases, such as cardiovascular and inflammatory diseases and cancer angiogenesis [2,3,4]. 

One of the most critical players of endothelial cells in the processes of vasculogenesis and angiogenesis is the growth factor VEGF [5]. Among the three existing receptors of VEGF, VEGFR-3 expression is restricted to lymphatic endothelial cells [6], while VEGFR-1 and VEGFR-2 are expressed in vascular endothelial cells [7]. Of the latter two, VEGFR1 has only a weak signaling potential and in fact acts as a decoy receptor via sequestration of VEGFA, thus acting as a negative regulator of VEGFR2 [7], while VEGFR-2 is the main receptor of VEGF and the main mediator of the vasculogenic and angiogenic properties of the vascular system [7,8,9]. Binding of VEGF to VEGFR2 results in its autophosphorylation, which leads to activation of downstream signaling molecules that control a number of cellular responses, such as the survival, migration, differentiation and proliferation of endothelial cells [7].

Growth factor receptors are not permanent residents of the cell surface; upon activation by their ligands, they become internalized via vesicular carriers that are generated at the plasma membrane. The internalized ligand–receptor complexes are directed to endosomes, and then the receptors are either guided to lysosomes for degradation or are recycled to the plasma membrane for another round of ligand binding, activation and internalization [10]. While the receptors undergo this trafficking process, they explore a complex network of endocytic compartments that mediate the intensity, duration and nature of downstream signaling cascades [11,12].

Depending on the individual receptor, internalization of the ligand–receptor complexes may take place through one or more different endocytic routes. Recent studies from our group showed that, in the presence of VEGF, as much as 70% of the internalized molecules of VEGFR2 are endocytosed via macropinocytosis, while the remaining 30% internalize through the clathrin-mediated route [13]. Internalization of the receptor through macropinocytosis is critical for downstream signaling, for endothelial cell functions and for angiogenesis in vivo [14]. However, depending on the specific cell type, VEGFR2 may explore additional pathways of entry, thereby controlling specific cellular functions via independent endocytic routes [15,16]. 

In a recent study, we tested the importance of clathrin- and dynamin-mediated endocytosis in VEGF signaling. We found that although clathrin or dynamin knockdown does not affect VEGF-induced activation of ERK1/2, dynasore, which is a chemical inhibitor of dynamin [17], besides exerting its specific effect in clathrin-mediated internalization of VEGFR2, causes strong inhibition of VEGF-mediated phosphorylation of ERK1/2 via an off-target effect (i.e., independently of its specific effect on endocytosis) [18]. 

In addition to dynasore, other dynamin inhibitors, i.e., dyngo 4a and dynole 34-2 [19], have been widely used to block dynamin-dependent endocytosis, especially when cells are resistant to transfection methods. However, since small molecule inhibitors may bind to multiple targets, it cannot be excluded that these inhibitors exert off-target effects [20,21], similarly to dynasore [18,22,23,24,25,26,27,28]. Here, by comparing the effect of the above drugs in VEGF-induced internalization of VEGFR2 and downstream signaling to ERK1/2 and Akt, we suggest that these inhibitors, besides exerting their specific effect in dynamin-dependent endocytosis, cause inhibitory effects on signaling that are independent of endocytosis; i.e., they are due to off-target effects.

## 2. Results

**Figure 1:**
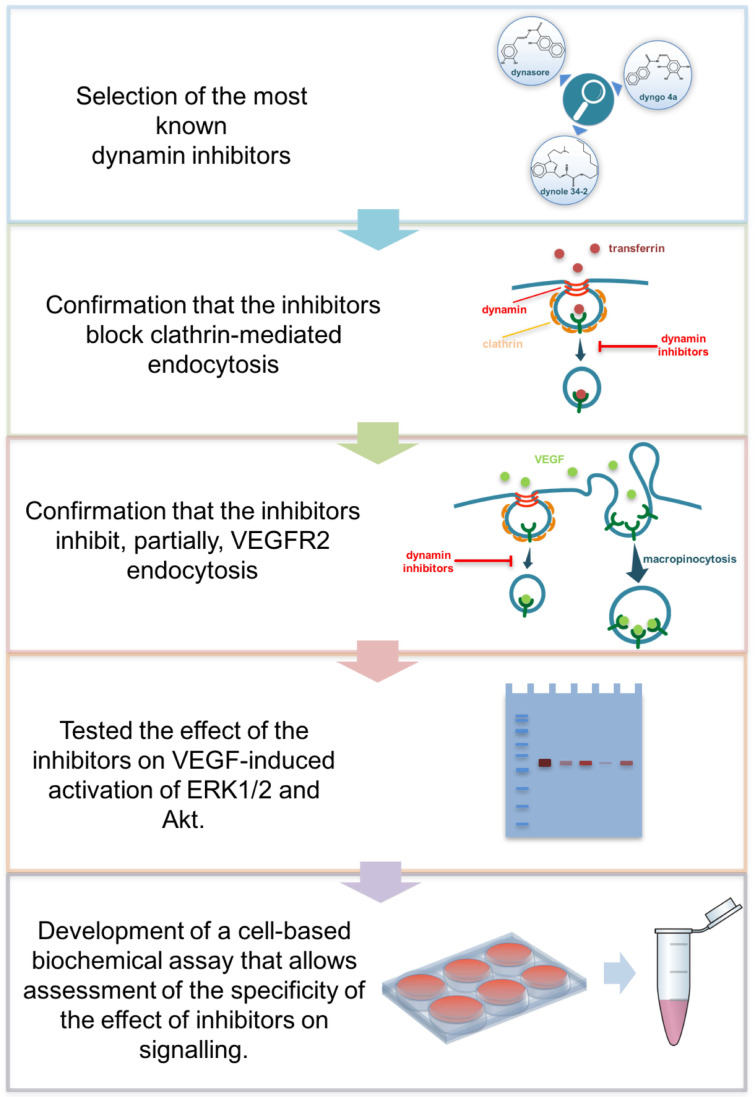
Overview of the workflow of this study. The figure outlines the experimental workflow used in the present study.

**Figure 2:**
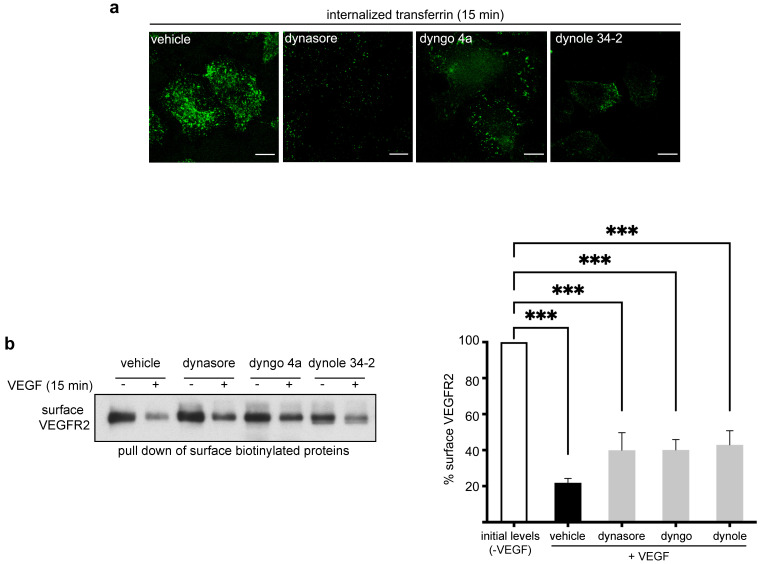
Chemical inhibitors of dynamin consistently block endocytosis of transferrin and inhibit partially the internalization of VEGFR2. (**a**) Serum-starved (2 h) HUVECs were treated with vehicle (DMSO) or dynasore (100 μM) or dyngo 4a (30 μM) or dynole 34-2 (20 μM) for 30 min. Subsequently, cells were incubated with FITC transferrin (15 min), fixed and processed for fluorescence microscopy analysis. Prior to fixation, membrane-bound transferrin was removed by acid wash (10 μm scale bars). (**b**) Analysis of VEGF-induced internalization of surface VEGFR2 through biotinylation of plasma membrane proteins. Serum-deprived HUVECs were treated for 30 min with vehicle or inhibitors of dynamin, i.e., dynasore, dyngo 4a or dynole 34-2, and incubated with VEGF for 15 min. Cells were transferred to 4 °C and surface proteins were labeled with cell-impermeable biotin. Surface biotinylated proteins were pulled down by streptavidin beads and processed for Western blotting analysis. Surface VEGFR2 was revealed by anti-VEGFR2 antibodies. Quantification of surface VEGFR2 is shown on the right of the Western blot (n = 3 independent experiments, mean ± S.E.M., *** *p* < 0.001, *t*-test).

In this study, we assessed the specificity of the most known dynamin inhibitors, i.e., dynasore [17], dyngo 4a [19] and dynole 34-2 [19], in VEGFR2 internalization and signaling. To this end, we tested their role in clathrin-mediated endocytosis (transferrin uptake), VEGFR2 internalization and VEGF-induced signaling to ERK1/2 and Akt (see workflow in Figure 1). At first, we confirmed that these drugs block clathrin-mediated endocytosis in endothelial cells. Indeed, dynasore and dynole 34-2 (Figure 2a), as well as dyngo 4a [18], substantially inhibit the internalization of fluorescently labeled transferrin in primary HUVECs. 

Subsequently, we assessed the effect of these drugs on VEGFR2 uptake. We found previously that, upon induction with VEGF, 30% of the molecules of VEGFR2 undergoing endocytosis are internalized through the clathrin-mediated pathway [14]. Here, using an internalization assay that is based on the pull-down of cell surface biotinylated proteins, we found that treatment of HUVECs with VEGF, for 15 min, led to internalization of 80% of the surface molecules of VEGFR2 (Figure 2b, vehicle-treated sample), while the inhibitors of dynamin inhibited internalization by 25% (Figure 2b). These data are consistent with previous reports showing that a fraction of VEGFR2 internalizes in a clathrin- and dynamin-dependent manner [14,18,29,30,31,32,33,34,35,36,37,38,39,40,41,42].

**Figure 3:**
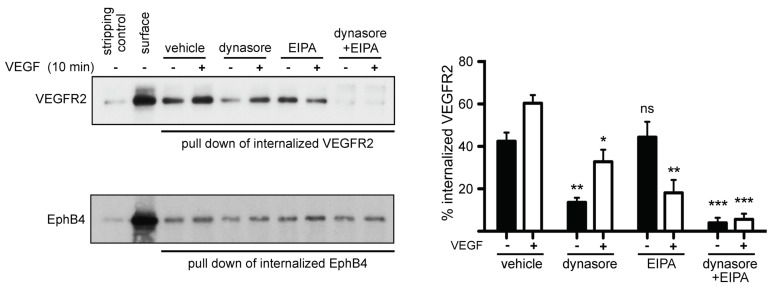
Analysis of VEGFR2 internalization routes, using biotinylation of plasma membrane proteins. Surface proteins of HUVECs were labeled at 4 °C with cell-impermeable, cleavable biotin. Then, the cells were treated with vehicle or inhibitors of endocytosis (dynasore, EIPA or dynasore + EIPA) for 30 min, followed by incubation in the presence or absence of VEGF at 37 °C for 10 min (to allow internalization). Surface biotin was cleaved, and internalized biotinylated proteins were pulled down using streptavidin beads and processed for Western blotting analysis. Internalized biotinylated VEGFR2 or biotinylated EphB4 was revealed by anti-VEGFR2 or anti-EphB4 antibodies, respectively. Quantification of internalized VEGFR2 is shown on the right of the immunoblot (n = 3 independent experiments, mean ± S.D., * *p* < 0.05, ** *p* < 0.01, *** *p* < 0.001, *t*-test).

Using a modified version of the above biotinylation assay, we confirmed that dynasore partially inhibits endocytosis of VEGFR2, while a larger fraction of VEGFR2 internalization is inhibited by EIPA (Figure 3), an inhibitor of macropinocytosis [43,44,45,46], consistently with previous findings [14]. The two drugs together block completely endocytosis of VEGFR2 (Figure 3), confirming that endocytosis of this receptor is exclusively accomplished by these two routes, i.e., clathrin-mediated endocytosis and macropinocytosis [14]. Altogether, the above findings show that the inhibitors of dynamin block the internalization of a fraction of surface molecules of VEGFR2, in agreement with earlier reports that documented the involvement of clathrin- and dynamin-dependent internalization of VEGFR2 [14,18,29,30,31,32,33,34,35,36,37,38,39,40,41,42].

**Figure 4:**
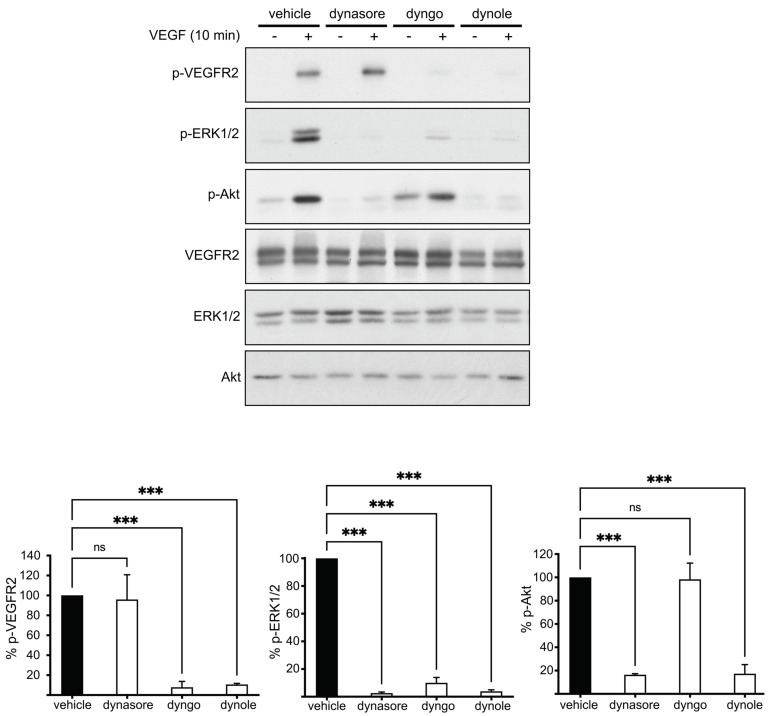
Chemical inhibitors of dynamin exert differential effects in VEGF signaling. Serum-starved HUVECs were subjected to 30 min treatment with vehicle or inhibitors of dynamin, followed by stimulation with VEGF (50 ng/mL) for 10 min. Subsequently, cells were lysed and were subjected to immunoblotting analysis using antibodies against the total or the phosphorylated protein forms of VEGFR2, ERK1/2 and Akt. Quantification of the effect of inhibitors on the VEGF-induced phosphorylation of VEGFR2 or ERK1/2 or Akt is shown on the right of the immunoblots (n = 3 independent experiments, mean ± S.E.M., *** *p* < 0.001, ANOVA). The Western blots corresponding to pERK1/2 and total ERK1/2 of vehicle- and dyngo-treated samples are reproduced from Figure S5a of our previous report [18], which falls under Creative Commons Attribution 4.0 International License (http://creativecommons.org/licenses/by/4.0/, accessed on 1 April 2021).

To assess the effect of dynamin inhibitors on VEGF signaling, we tested their impact on VEGF-mediated activation of VEGFR2 and downstream signaling kinases. Surprisingly, the inhibitors of dynamin exerted differential effects on VEGF-induced phosphorylation of VEGFR2 and on the downstream kinases ERK1/2 and Akt (Figure 4). More specifically, although dynasore had no effect on VEGFR2 phosphorylation, dyngo 4a and dynole caused strong inhibition (Figure 4). On the other hand, dynasore and dynole inhibited Akt phosphorylation, while dyngo had no effect (Figure 4). As dynamin inhibitors have a consistent inhibitory effect on the internalization of VEGFR2 (see previous paragraph), the above contradictory effects of the drugs in VEGF-induced phosphorylation of signaling molecules suggest that their effect on signaling is independent of their impact in endocytosis; i.e., it is due to off-target effects. This conclusion is consistent with earlier reports showing that clathrin- or dynamin-dependent endocytosis is not essential for VEGF-induced signaling [14,18,31,32,34,38,39] and that dynasore exerts an off-target inhibitory effect on VEGF-induced phosphorylation of ERK1/2 [18].

**Figure 5:**
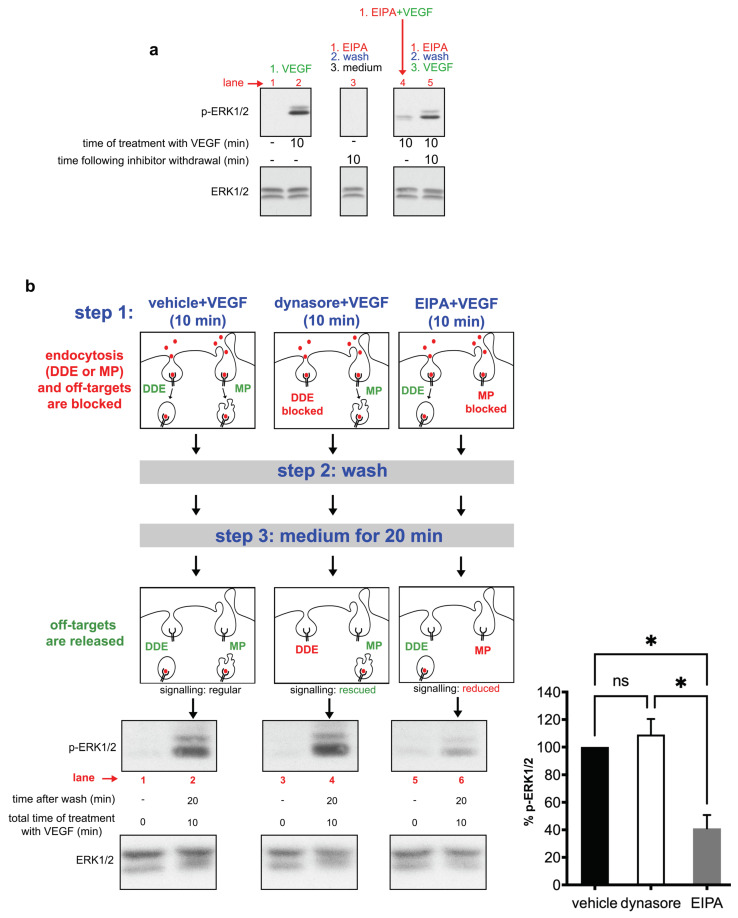
Comparative validation of the specificity of two endocytic inhibitors (dynasore and EIPA) in VEGF-induced phosphorylation of ERK1/2. (**a**) EIPA-mediated inhibition of ERK1/2 phosphorylation is reversible. Serum-deprived HUVECs were treated in different steps, as shown above the lanes of the blots. The first two lanes show control samples of vehicle-treated (lane 1) or VEGF-treated (lane 2) cells. In lane 5, HUVECs were treated first with EIPA for 30 min (step 1, EIPA), washed and incubated in serum-free medium for 10 min to allow recovery from the drug (step 2, wash) and stimulated with VEGF (step 3, VEGF). To test that the reversibility of the effect of the drug still depends on activation by VEGF, an identically treated sample (as above), but without VEGF, was analyzed in parallel (lane 4). The arrow indicates a sample that was stimulated in the presence of the inhibitor to control the effectiveness of the drug (lane 4). Following the final treatment step, all samples were subjected to immunoblotting analysis using antibodies against phosphorylated ERK1/2. Sample loading was assessed by the total protein levels of ERK1/2. (**b**) Employment of the “uncoupling experiment“ to comparatively validate the specificity of dynasore and EIPA in VEGF-induced phosphorylation of ERK1/2. HUVECs were treated in three steps, as indicated in blue font. In step 1, cells were treated with vehicle + VEGF, dynasore + VEGF or EIPA + VEGF, for 10 min. Inside the schemes, the active pathways for each condition are indicated in green font and the inactive pathways are indicated in red font. On the left side of the top schemes, the text indicates that the drugs block both endocytosis and the off-targets. In step 2, the cells were acid washed to remove membrane-bound VEGF and the drug. The effectiveness of the acid wash was confirmed in Supplementary Figure S1. In step 3, the cells were incubated in serum-free medium, for 20 min, to allow full recovery, thereby releasing possible off-target effects of the drug (indicated on the left side of the schemes as “off-targets are released”, in green font). As there was no ligand in this step, DDE and MP could not resume when the samples had been treated (in step 1) with dynasore or EIPA, respectively, despite removal of the drug. Blocked pathways are indicated in the schemes with red font, while the active ones are indicated in green font. Thus, DDE (lane 4) and MP (lane 6) did not take place throughout the whole experiment, even after removal of the drug (note that this is the main difference from the reversibility experiment shown in Figure 5a, where VEGF was added after the removal of the drug, thereby rescuing endocytosis of VEGFR2). Finally, the cells were lysed and subjected to immunoblotting analysis using anti-phospho-ERK1/2 antibodies. Since DDE never took place in sample 4 (see above), recovery of the signal in this sample (compare lane 4 with lane 2) upon release of the off-targets suggests that DDE is not essential for VEGF signaling; thus, the inhibitory effect of dynasore shown in Figure 4 is due to the off-targets. On the other hand, in sample 6 (where MP was inhibited), the signal did not recover despite removal of the drug (compare lane 6 with lane 2), which releases possible off-targets, thus suggesting that MP is essential for VEGF signaling. Quantification is shown on the right of the immunoblots (n = 3 independent experiments, mean ± S.E.M., * *p* < 0.05, ANOVA).

Given that endocytic inhibitors are extensively used for testing the role of endocytosis in signal transduction pathways, the above findings suggest that it is critical to evaluate whether the effect of a drug on signaling is specifically due to its impact on endocytosis or due to off-targets. To this end, we have recently developed a cell-based protocol called “uncoupling assay”, owing to its ability to uncouple the specific inhibitory effect of a drug in trafficking from the putative off-target effects on signaling molecules [18]. Here, we used this assay to comparatively validate the specificity of the effect of two endocytic inhibitors, dynasore versus EIPA, in VEGF-to-ERK1/2 activation. For reasons that will become evident below, it is critical to test, at first, whether the effects of the drugs on signaling are reversible. We have already shown the reversibility of the inhibitory effect of dynasore in VEGF-induced phosphorylation of ERK [18]; that is, we found that VEGF-induced activation of ERK is totally rescued after removal of the drug. Here, we tested whether the same is true for EIPA, the inhibitor of macropinocytosis. As shown in Figure 5a, VEGF-induced ERK1/2 phosphorylation is inhibited by EIPA (compare lanes 2 and 4 in Figure 5a), while withdrawal of the drug followed by further incubation with VEGF resulted in an almost complete rescue of ERK1/2 phosphorylation (lane 5). These data suggest that, similarly to dynasore [18], the inhibitory effect of EIPA is reversible upon withdrawal of the drug. 

We then proceeded in using the “uncoupling experiment” to compare the specificity of these two drugs in VEGF-induced phosphorylation of ERK1/2. The uncoupling experiment consisted of three steps (drawn schematically in detail in Figure 5b). In step 1, HUVECs were treated for 10 min with VEGF + vehicle, VEGF + dynasore (to prevent dynamin-dependent endocytosis, DDE) or VEGF + EIPA (to inhibit macropinocytosis (MP)). As clathrin-mediated endocytosis and macropinocytosis are the main routes of internalization of VEGFR2 [14], treatment with dynasore inhibits both DDE and possible off-targets, while the receptor can still internalize through MP. On the other hand, EIPA inhibits both MP and possible off-targets, while the receptor can still internalize through DDE. The schemes in Figure 5 depict the different pathways of entry of VEGFR2, namely dynamin-dependent endocytosis (DDE) and micropinocytosis (MP), as well as the steps at which the off-targets were inhibited or released. In step 2, the cells were twice acid-washed to remove any remaining molecules of VEGF from the plasma membrane, followed by three washes with saline buffer to withdraw the drug. The effectiveness of the acid wash was confirmed, as shown in Supplementary Figure S1, consistently with earlier studies [17,47]. Subsequently, in step 3, the samples were incubated in plain medium for 20 min to release possible off-target effects of the drugs. Note that, in this last step (step 3), given that there was no ligand in the medium, DDE and MP could not resume despite removal of the drugs (indicated in the scheme as “DDE” or “MP” in red font), while the off-targets were released (indicated in the scheme as “off-targets are released” in green font). Thus, DDE and MP did not take place throughout the whole experiment in lanes 4 and 6, respectively, even after removal of the drug. As DDE never took place in sample 4, and the signal was comparable to vehicle-treated cells (compare lane 4 with lane 2), it is concluded that clathrin- or dynamin-dependent endocytosis is not essential for VEGF signaling, consistently with previous studies [14,18,31,32,34,38,39]. Thus, the inhibitory effect of dynasore in ERK phosphorylation (Figure 4) is due to an off-target effect [18]. In cells treated with VEGF + EIPA, where the receptor is not allowed to enter via macropinocytosis, the phosphorylation of ERK was substantially inhibited (compare lane 6 with control lane 2), despite removal of the drug, which releases putative off-targets. Thus, the inhibitory effect of EIPA was not due to an off-target effect; it was rather due to the inhibitory effect of EIPA during the first step (inhibition of trafficking). These data are in line with previous findings showing a crucial role of macropinocytosis in VEGF-mediated phosphorylation of ERK1/2 [14]. Altogether, these data suggest that the inhibitory effect of dynasore in VEGF-induced activation of ERK1/2 is due to an off-target effect on signaling, in agreement with a previous study [18], while the effect of EIPA is specifically due to inhibition of macropinocytosis.

## 3. Discussion

Among the different endocytic pathways that have been described so far, clathrin-mediated endocytosis (CME) is the most well known [48]. In this pathway, the large GTPase dynamin is responsible for catalyzing the last step of the generation of clathrin-coated vesicles, i.e., the pinching of the clathrin-coated pits from the plasma membrane [48,49]. Due to the central role of dynamin in mediating CME [50], as well as to its additional role in other key cellular functions [48,50,51,52], a number of small-molecule inhibitors targeting its activity have been generated [17,19,53,54]. These tools have proven to be valuable in studying the role of dynamin in a large number of cellular functions [20,21], especially in difficult-to-transfect cells [55]. As they offer the advantage of a rapid effect, these drugs avoid the manifestation of compensatory trafficking pathways [56]. However, despite their wide use and their undoubtful inhibitory effect on dynamin, the fact that they are small molecules that could bind to multiple targets raises concerns about possible off-target effects [18,20,21,22,23,24,25,26,27,28]. The most compelling evidence in favor of this concern comes from experiments in dynamin triple knockout cells, where, although lack of all three dynamins did not affect fluid-phase endocytosis and membrane ruffling, dynasore and dyngo robustly inhibited these two processes both in wild-type and in the triple knockout cells [22].

Here, we compared the specificity of the effects of three dynamin inhibitors (dynasore, dyngo and dynole) on VEGFR2 internalization and signaling to ERK1/2 and Akt. Surprisingly, although these drugs consistently inhibited clathrin-mediated endocytosis of both transferrin and VEGFR2, they exerted differential effects on VEGF-induced phosphorylation of VEGFR2 and Akt (Figure 4 and [18]). More specifically, although dynasore had no effect on VEGF-induced phosphorylation of VEGFR2, the other two drugs caused strong inhibition. Additionally, despite the fact that dyngo did not interfere with VEGF-induced phosphorylation of Akt, dynasore and dynole had a strong inhibitory action. These contradictory effects between the three dynamin inhibitors suggest that although these drugs are specific in inhibiting CME of transferrin and VEGFR2 (Figure 2), their effects in VEGF-induced signaling are unrelated to inhibition of endocytosis; i.e., they are due to off-target effects. This conclusion is in complete agreement with previous reports showing that clathrin- and dynamin-mediated endocytosis of VEGFR2 is not essential for VEGF-induced signaling and angiogenesis [14,18,31,32,34,38,39]. The off-targets of these drugs could be either molecules playing a direct role in VEGF signaling (e.g., Ras, Raf or PI3K) or molecules that may indirectly affect the correct positioning and/or activity of the VEGF signaling machinery (e.g., cytoskeletal molecules or trafficking regulators).

The present study underscores the importance of using multiple endocytic inhibitors/drugs against a molecule or pathway when addressing its role in functional assays. Using this approach, when all drugs exert the same effect, it is highly probable that this effect is specific. On the other hand, when they have contrasting effects, it is likely that these effects are unrelated to endocytosis. Other complementary approaches, such as knockdown techniques, when cells are easy to transfect, are valuable in assessing the specificity of the conclusions drawn from drug-based experiments. A recently developed alternative approach assesses whether the effect of a drug on signaling is solely and specifically due to inhibition of endocytosis or due to an off-target effect [18]. Here, using this assay, we compared the specificity of two endocytic inhibitors, dynasore and EIPA. Interestingly, we found that although both dynasore and EIPA inhibit ERK1/2 phosphorylation, the effect of dynasore is unrelated to inhibition of dynamin-dependent endocytosis (that is, it is due to an off-target effect), while the effect of EIPA in this assay is due to its specific effect in inhibiting macropinocytosis (it cannot be excluded though that EIPA, in other cellular functions, may also exert unspecific effects). These data are consistent with previous studies showing that clathrin- and dynamin-mediated endocytosis is not essential in VEGF-induced signaling [14,18,31,32,34,38,39], while macropinocytosis is crucial [14]. This protocol could thus be applied in the study of other signaling pathways and/or other cellular functions to address whether the effect of a drug on signaling is solely due to inhibition of endocytosis or due to off-target blockage.

## 4. Materials and Methods

### 4.1. Reagents and Antibodies

The concentration of the reagents used in this study, unless stated otherwise, is indicated below in parentheses. Recombinant human VEGF_165_ (50 ng/mL) was obtained from Immunotools GmbH, Friesoythe, Germany. Dynasore (100 µmol/L) and 5-N-ethyl-N-isopropyl amiloride (EIPA) (50 µmol/L) were from Sigma-Aldrich/Merck KGaA, Darmstadt, Germany, whereas dyngo 4a and dynole 34-2 were from Abcam plc, Cambridge, UK. The antibodies against ERK1/2, p-ERK1/2, Akt, p-Akt, VEGFR2 (55B11) and p-VEGFR2 (tyr1175) (19A10) were from Cell Signaling Technology, Danvers, MA. The antibodies against EphB4 were from R&D Systems R&D Systems Inc., Minneapolis, MN. The HRP-conjugated antibodies used in Western blotting experiments were from Jackson Immunoresearch Europe Ltd., Cambridge, UK. All other reagents used in the present study were obtained from Merck/Sigma, Darmstadt, Germany, unless stated otherwise.

### 4.2. Endothelial Cell Culture

Human umbilical vein endothelial cells (HUVECs) were cultured as described previously [57,58]. Briefly, HUVECs were seeded on 100 mm dishes coated with collagen type I and were cultured in M199 medium supplemented with 20% fetal calf serum, 1% penicillin–streptomycin, 0.05 mg/mL endothelial cell growth supplement and 0.05 IU/mL heparin until they formed confluent cell monolayers. Then, cells were re-seeded in 24-well plates and treated according to the needs of the different assays.

### 4.3. Direct Treatment of Cells with the Inhibitors

All VEGF-dependent experiments were carried out using 2 h serum-deprived HUVECs, followed by a 30 min pretreatment step with vehicle or inhibitors. Drug treatments were carried out in serum-free M199 medium. For the VEGF-independent experiments, HUVECs were treated with vehicle (DMSO) or with the drug for various durations, lysed in PBS supplemented with 1% SDS and analyzed by Western blotting using rabbit anti-VEGFR2 cytoplasmic domain antibodies (Cell Signaling Technology, #2479S) or other antibodies as indicated.

### 4.4. Reversible Inhibition of Drug Treatment

To test the reversibility of the inhibitory effect of EIPA in VEGFR2 signaling, serum-deprived cells (2 h) were treated with EIPA for 30 min. Following this treatment, the cells were washed 3 times with Ca^2+^/Mg^2+^ HBSS, followed by incubation in serum-free M199 medium for 10 min. Then, the cells were stimulated with VEGF for 10 min, lysed and analyzed by immunoblotting using antibodies against ERK1/2 and phosphorylated ERK1/2. Vehicle-treated cells were treated as above.

### 4.5. Uncoupling Experiment

This protocol consisted of three steps. During step 1, serum-starved HUVECs (2 h) were treated with vehicle or inhibitors of endocytosis (dynasore or EIPA) for 30 min, followed by stimulation with VEGF (50 ng/mL) for 10 min in the presence of the above drugs. Then, the cells were processed further to rescue the off-target effects of the drugs against signaling molecules. To this end, in step 2, cells were twice acid-washed (M199 medium, pH 2.0) to remove any remaining molecules of VEGF from the plasma membrane, followed by 3 washes with Ca^2+^/Mg^2+^ HBSS. The effectiveness of the acid wash was confirmed, as shown in Supplementary Figure S1, consistently with earlier studies [17,47]. Then (in step 3), the cells were incubated in fresh serum-free medium for 20 min (this incubation rescues any off-target effects of the drug), lysed and analyzed by Western blotting using antibodies against ERK1/2 or phosphorylated ERK1/2. 

### 4.6. Microscopy-Based Internalization Assay

HUVECs were cultured in plastic dishes that were obtained from ibidi, 35 mm round, appropriate for microscopy, previously coated with collagen type I. For internalization experiments, cells were washed 3 times with blocking buffer, treated for 30 min with vehicle or inhibitors in blocking buffer, transferred to 37 °C and incubated with 50 μg/mL fluorescein isothiocyanate-conjugated transferrin (Invitrogen). Then, the samples were washed 3 times with Ca^2+^/Mg^2+^ HBSS, fixed in 3.7% PFA for 20 min and analyzed by microscopy. Images of the cells were captured using a Leica TCS SP5 II scanning confocal microscope and a Leica 63X HCX PL APO 1.4 NA objective. Imaging data were subsequently processed using the LAS AF software, according to the manufacturer’s guidelines.

### 4.7. Biotinylation-Based Internalization Assays

The amount of surface or internalized VEGFR2, or internalized EphB4, was assessed biochemically in serum-starved HUVECs (2 h). Following starvation, the cells were transferred to 4 °C and incubated with 0.5 mg/mL cleavable EZ-Link Sulfo-NHS-S-S-Biotin (Thermo-Scientific) in Ca^2+^/Mg^2+^ HBSS for 20 min to label cell surface proteins. Then, the samples were washed 2 times with 50 mM glycine in PBS, to quench unbound biotin, and treated for 30 min with vehicle or inhibitors in serum-free M199 medium supplemented with 20 mmol/L HEPES. Cells were shifted to prewarmed media (in the presence or absence of VEGF and inhibitors) and transferred to 37 °C for 10 min. Then, cells were transferred back to 4 °C and incubated 2 × 15 min with biotin cleavage buffer (100 mM sodium 2-mercaptoethanesulfonate, MESNA, 50 mM Tris pH 8.5, 100 mM NaCl, 1 mM EDTA, 0.1% BSA) to strip biotin from the noninternalized proteins remaining at the cell surface. MESNA was quenched with 50 mM iodoacetamide in PBS for 10 min, and cells were lysed in lysis buffer (0.5% Triton X-100, 0.5% NP-40, 50 mM Tris pH 7.5, 100 mM NaCl, 5 mM EDTA and Roche protease inhibitor cocktail). Finally, the samples were processed using standard pull-down protocol with streptavidin beads. Control samples were treated at 4 °C to analyze the surface levels of VEGFR2, or EphB4, of nonstimulated cells and the efficiency of biotin cleavage. Prior to all treatments at 4 °C, the cell samples were washed 3 times with ice-cold Ca^2+^/Mg^2+^ HBSS.

For the analysis of VEGFR2 uptake from the plasma membrane, serum-starved HUVECs were treated for 30 min with vehicle or inhibitors, stimulated with VEGF for 15 min, transferred to 4 °C and labeled with biotin, as described above. Following biotin quenching, cells were lysed and processed by pull-down using streptavidin beads.

### 4.8. Quantification and Statistical Analysis

The quantification of immunoblots was performed using the ImageJ software. The values reported in the figures represent means ± S.E.M. or S.D. calculated from at least 3 replicates for each experimental setting. Statistical differences were evaluated using the Student’s t-test for two-group comparison or analysis of variance (ANOVA) followed by Dunnett’s post hoc analysis for comparisons of more than two groups.

## Data Availability

Data is contained within the article or supplementary material.

## Supplementary Materials

The following are available online at https://www.mdpi.com/article/10.3390/cells10050997/s1, Figure S1: Assessment of the efficiency of acid wash in stripping surface bound anti-VEGFR2 antibodies.

## References

[1] Tang D.G., Conti C.J. (2004). Endothelial Cell Development, Vasculogenesis, Angiogenesis, and Tumor Neovascularization: An Update. Semin. Thromb. Hemost..

[2] McGill S.N., Ahmed N.A., Christou N.V. (1998). Endothelial cells: Role in infection and inflammation. World J. Surg..

[3] Cines D.B., Pollak E.S., Buck C.A., Loscalzo J., Zimmerman G.A., McEver R.P., Pober J.S., Wick T.M., Konkle B.A., Schwartz B.S. (1998). Endothelial cells in physiology and in the pathophysiology of vascular disorders. Blood.

[4] Eilken H.M., Adams R.H. (2010). Dynamics of endothelial cell behavior in sprouting angiogenesis. Curr. Opin. Cell Biol..

[5] Coultas L., Chawengsaksophak K., Rossant J. (2005). Endothelial cells and VEGF in vascular development. Nat. Cell Biol..

[6] Joukov V., Pajusola K., Kaipainen A., Chilov D., Lahtinen I., Kukk E., Saksela O., Kalkkinen N., Alitalo K. (1996). A novel vascular endothelial growth factor, VEGF-C, is a ligand for the Flt4 (VEGFR-3) and KDR (VEGFR-2) receptor tyrosine kinases. EMBO J..

[7] Olsson A.-K., Dimberg A., Kreuger J., Claesson-Welsh L. (2006). VEGF receptor signalling in control of vascular function. Nat. Rev. Mol. Cell Biol..

[8] Carmeliet P. (2005). Angiogenesis in life, disease and medicine. Nat. Cell Biol..

[9] Holmes K., Roberts O.L., Thomas A.M., Cross M.J. (2007). Vascular endothelial growth factor receptor-2: Structure, function, intracellular signalling and therapeutic inhibition. Cell. Signal..

[10] Platta H.W., Stenmark H. (2011). Endocytosis and signaling. Curr. Opin. Cell Biol..

[11] Gonzalez-Gaitan M., Stenmark H. (2003). Endocytosis and signaling: A relationship under development. Cell.

[12] Miaczynska M., Christoforidis S., Giner A., Shevchenko A., Uttenweiler-Joseph S., Habermann B., Wilm M., Parton R.G., Zerial M. (2004). APPL Proteins Link Rab5 to Nuclear Signal Transduction via an Endosomal Compartment. Cell.

[13] Basagiannis D., Christoforidis S. (2016). Constitutive Endocytosis of VEGFR2 Protects the Receptor against Shedding. J. Biol. Chem..

[14] Basagiannis D., Zografou S., Murphy C., Fotsis T., Morbidelli L., Ziche M., Bleck C., Mercer J., Christoforidis S. (2016). VEGF induces signalling and angiogenesis by directing VEGFR2 internalisation via macropinocytosis. J. Cell Sci..

[15] Simons M., Gordon E., Claesson-Welsh E.G.L. (2016). Mechanisms and regulation of endothelial VEGF receptor signalling. Nat. Rev. Mol. Cell Biol..

[16] Genet G., Boyé K., Mathivet T., Ola R., Zhang F., Dubrac A., Li J., Genet N., Geraldo L.H., Benedetti L. (2019). Endophilin-A2 dependent VEGFR2 endocytosis promotes sprouting angiogenesis. Nat. Commun..

[17] Macia E., Ehrlich M., Massol R., Boucrot E., Brunner C., Kirchhausen T. (2006). Dynasore, a Cell-Permeable Inhibitor of Dynamin. Dev. Cell.

[18] Basagiannis D., Zografou S., Galanopoulou K., Christoforidis S. (2017). Dynasore impairs VEGFR2 signalling in an endocytosis-independent manner. Sci. Rep..

[19] Robertson M.J., Deane F.M., Robinson P.J., McCluskey A. (2014). Synthesis of Dynole 34-2, Dynole 2-24 and Dyngo 4a for investigating dynamin GTPase. Nat. Protoc..

[20] Ivanov A.I. (2014). Pharmacological Inhibitors of Exocytosis and Endocytosis: Novel Bullets for Old Targets. Adv. Struct. Saf. Stud..

[21] Ivanov A.I. (2008). Pharmacological Inhibition of Endocytic Pathways: Is It Specific Enough to Be Useful?. Methods Mol. Biol..

[22] Park R.J., Shen H., Liu L., Liu X., Ferguson S.M., De Camilli P. (2013). Dynamin triple knockout cells reveal off target effects of commonly used dynamin inhibitors. J. Cell Sci..

[23] Preta G., Lotti V., Cronin J.G., Sheldon I.M. (2015). Protective role of the dynamin inhibitor Dynasore against the cholesterol-dependent cytolysin of Trueperella pyogenes. FASEB J..

[24] Preta G., Cronin J.G., Sheldon I.M. (2015). Dynasore—not just a dynamin inhibitor. Cell Commun. Signal..

[25] Persaud A., Cormerais Y., Pouyssegur J., Rotin D., Avinash P., Yann C., Jacques P., Daniela R. (2018). Dynamin inhibitors block activation of mTORC1 by amino acids independently of dynamin. J. Cell Sci..

[26] Webster A., Chintala S.K., Kim J., Ngan M., Itakura T., Panjwani N., Argüeso P., Barr J.T., Jeong S., Fini M.E. (2018). Dynasore protects the ocular surface against damaging oxidative stress. PLoS ONE.

[27] Jiang Z., He H., Liu H., Thayumanavan S. (2019). Cellular Uptake Evaluation of Amphiphilic Polymer Assemblies: Importance of Interplay between Pharmacological and Genetic Approaches. Biomacromolecules.

[28] Clemente L.P., Rabenau M., Tang S., Stanka J., Cors E., Stroh J., Culmsee C., Von Karstedt S. (2020). Dynasore Blocks Ferroptosis through Combined Modulation of Iron Uptake and Inhibition of Mitochondrial Respiration. Cells.

[29] Bhattacharya R., Kang-Decker N., Hughes D.A., Mukherjee P., Shah V., McNiven M.A., Mukhopadhyay D. (2005). Regulatory role of dynamin-2 in VEGFR-2/KDR-mediated endothelial signaling. FASEB J..

[30] Ewan L.C., Jopling H.M., Jia H., Mittar S., Bagherzadeh A., Howell G.J., Walker J.H., Zachary I.C., Ponnambalam S. (2006). Intrinsic tyrosine kinase activity is required for vascular endothelial growth factor receptor 2 ubiquitination, sorting and degradation in endothelial cells. Traffic.

[31] Lampugnani M.G., Orsenigo F., Gagliani M.C., Tacchetti C., Dejana E. (2006). Vascular endothelial cadherin controls VEGFR-2 internalization and signaling from intracellular compartments. J. Cell Biol..

[32] Bruns A.F., Herbert S.P., Odell A.F., Jopling H.M., Hooper N.M., Zachary I.C., Walker J.H., Ponnambalam S. (2009). Ligand-Stimulated VEGFR2 Signaling is Regulated by Co-Ordinated Trafficking and Proteolysis. Traffic.

[33] Sawamiphak S., Seidel S., Essmann C.L., Wilkinson G.A., Pitulescu M.E., Acker T., Acker-Palmer A. (2010). Ephrin-B2 regulates VEGFR2 function in developmental and tumour angiogenesis. Nat. Cell Biol..

[34] Pasula S., Cai X., Dong Y., Messa M., McManus J., Chang B., Liu X., Zhu H., Mansat R.S., Yoon S.-J. (2012). Endothelial epsin deficiency decreases tumor growth by enhancing VEGF signaling. J. Clin. Investig..

[35] Bruns A.F., Yuldasheva N., Latham A.M., Bao L., Pellet-Many C., Frankel P., Stephen S.L., Howell G.J., Wheatcroft S.B., Kearney M.T. (2012). A Heat-Shock Protein Axis Regulates VEGFR2 Proteolysis, Blood Vessel Development and Repair. PLoS ONE.

[36] Gourlaouen M., Welti J.C., Vasudev N.S., Reynolds A.R. (2013). Essential Role for Endocytosis in the Growth Factor-stimulated Activation of ERK1/2 in Endothelial Cells. J. Biol. Chem..

[37] Nakayama M., Nakayama A., Van Lessen M., Yamamoto H., Hoffmann S., Drexler H.C.A., Itoh N., Hirose T., Breier G., Vestweber D. (2013). Spatial regulation of VEGF receptor endocytosis in angiogenesis. Nat. Cell Biol..

[38] Tessneer K.L., Pasula S., Cai X., Dong Y., McManus J., Liu X., Yu L., Hahn S., Chang B., Chen Y. (2014). Genetic Reduction of Vascular Endothelial Growth Factor Receptor 2 Rescues Aberrant Angiogenesis Caused by Epsin Deficiency. Arter. Thromb. Vasc. Biol..

[39] Lee M.Y., Skoura A., Park E.J., Landskroner-Eiger S., Jozsef L., Luciano A.K., Murata T., Pasula S., Dong Y., Bouaouina M. (2014). Dynamin 2 regulation of integrin endocytosis, but not VEGF signaling, is crucial for developmental angiogenesis. Development.

[40] Okabe K., Kobayashi S., Yamada T., Kurihara T., Tai-Nagara I., Miyamoto T., Mukouyama Y.-S., Sato T.N., Suda T., Ema M. (2014). Neurons Limit Angiogenesis by Titrating VEGF in Retina. Cell.

[41] Koch S., van Meeteren L.A., Morin E., Testini C., Weström S., Björkelund H., Le Jan S., Adler J., Berger P., Claesson-Welsh L. (2014). NRP1 Presented in trans to the Endothelium Arrests VEGFR2 Endocytosis, Preventing Angiogenic Signaling and Tumor Initiation. Dev. Cell.

[42] Salikhova A., Wang L., Lanahan A.A., Liu M., Simons M., Leenders W.P.J., Mukhopadhyay D., Horowitz A. (2008). Vascular Endothelial Growth Factor and Semaphorin Induce Neuropilin-1 Endocytosis via Separate Pathways. Circ. Res..

[43] Kerr M.C., Teasdale R.D. (2009). Defining Macropinocytosis. Traffic.

[44] Koivusalo M., Welch C., Hayashi H., Scott C.C., Kim M., Alexander T., Touret N., Hahn K.M., Grinstein S. (2010). Amiloride inhibits macropinocytosis by lowering submembranous pH and preventing Rac1 and Cdc42 signaling. J. Cell Biol..

[45] Kühling L., Schelhaas M. (2014). Systematic Analysis of Endocytosis by Cellular Perturbations. Methods Mol. Biol..

[46] Commisso C., Flinn R.J., Bar-Sagi D. (2014). Determining the macropinocytic index of cells through a quantitative image-based assay. Nat. Protoc..

[47] Kirchhausen T., Macia E., Pelish H.E. (2008). Use of Dynasore, the Small Molecule Inhibitor of Dynamin, in the Regulation of Endocytosis. Methods Enzymol..

[48] McMahon H.T., Boucrot E. (2011). Molecular mechanism and physiological functions of clathrin-mediated endocytosis. Nat. Rev. Mol. Cell Biol..

[49] Ferguson S.M., De Camilli P. (2012). Dynamin, a membrane-remodelling GTPase. Nat. Rev. Mol. Cell Biol..

[50] Schmid S.L., Frolov V.A. (2011). Dynamin: Functional Design of a Membrane Fission Catalyst. Annu. Rev. Cell Dev. Biol..

[51] Sever S., Chang J., Gu C. (2013). Dynamin Rings: Not Just for Fission. Traffic.

[52] Kaksonen M., Roux A. (2018). Mechanisms of clathrin-mediated endocytosis. Nat. Rev. Mol. Cell Biol..

[53] McCluskey A., Daniel J.A., Hadzic G., Chau N., Clayton E.L., Mariana A., Whiting A., Gorgani N.N., Lloyd J.R., Quan A. (2013). Building a Better Dynasore: The Dyngo Compounds Potently Inhibit Dynamin and Endocytosis. Traffic.

[54] Quan A., McGeachie A.B., Keating D.J., Van Dam E.M., Rusak J., Chau N., Malladi C.S., Chen C., McCluskey A., Cousin M.A. (2007). Myristyl Trimethyl Ammonium Bromide and Octadecyl Trimethyl Ammonium Bromide Are Surface-Active Small Molecule Dynamin Inhibitors that Block Endocytosis Mediated by Dynamin I or Dynamin II. Mol. Pharmacol..

[55] Sadowski Ł., Jastrzębski K., Kalaidzidis Y., Heldin C., Hellberg C., Miaczynska M. (2013). Dynamin Inhibitors Impair Endocytosis and Mitogenic Signaling of PDGF. Traffic.

[56] Damke H., Baba T., Van Der Bliek A.M., Schmid S.L. (1995). Clathrin-independent pinocytosis is induced in cells overexpressing a temperature-sensitive mutant of dynamin. J. Cell Biol..

[57] Papanikolaou A., Papafotika A., Christoforidis S. (2011). CD39 Reveals Novel Insights into the Role of Transmembrane Domains in Protein Processing, Apical Targeting and Activity. Traffic.

[58] Zografou S., Basagiannis D., Papafotika A., Shirakawa R., Horiuchi H., Auerbach D., Fukuda M., Christoforidis S. (2012). A complete Rab screening reveals novel insights in Weibel-Palade body exocytosis. J. Cell Sci..

