# Supplementary material for: Chemical Inhibitors of Dynamin Exert Differential Effects in VEGF Signaling
Source: Cells. 2021 Apr 23;10(5):997. doi: 10.3390/cells10050997 (PMC8145957; doi:10.3390/cells10050997)
Supplement: Supplementary file 1 [file cells-10-00997-s001.zip › cells-1188271-supplementary.pdf]

Supplementary figure 1

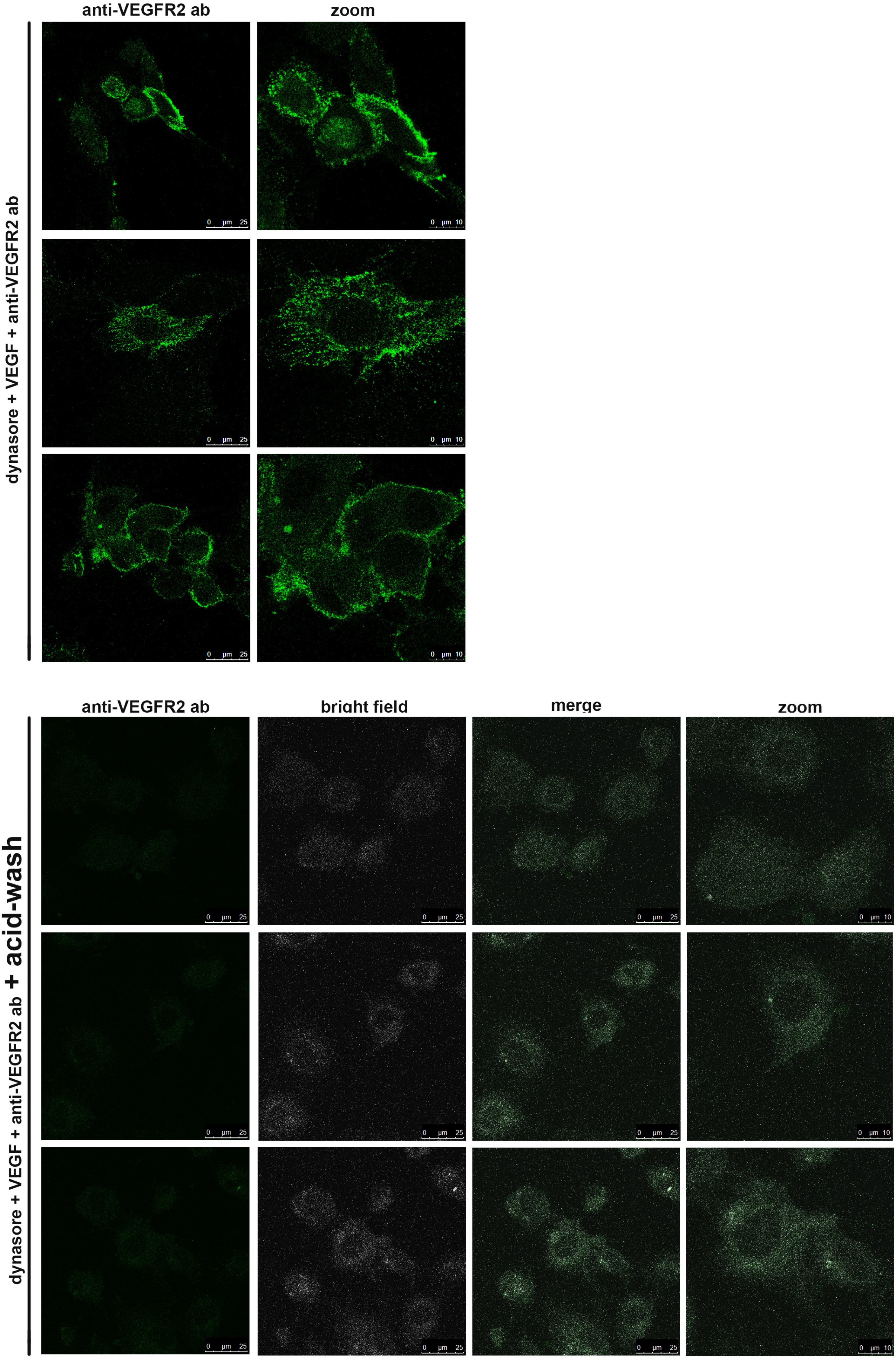

Supplementary figure 1. Assessment of the efficiency of acid wash in stripping surface bound anti-VEGFR2 antibodies. To assess the effectiveness of acid wash in the uncoupling experiment of Fig. 5, serum starved HUVECs were treated with dynasore (100 μM) for 10 min (to block clathrin-mediated endocytosis), followed by stimulation with VEGF (50 ng/ml), in the presence of dynasore and antibodies against the extracellular domain of VEGFR2 (ab9530) (10 μg/ml) for 10 min (the antibodies were used as a probe to assess the effectiveness of the acid wash). Cells were then washed with control medium (upper three examples of fields), or acid-washed (bottom three examples of fields), fixed and processed for immunofluorescence microscopy analysis (in the absence of permeabilization) to reveal surface-bound anti-VEGFR2 antibodies, using secondary antibodies labeled with Alexa-488. In the case of the acid-washed samples, as the signal of the antibodies is too weak, bright field was used to reveal the position of the cells (gray). Note that the presence of dynasore in this experiment allows testing of the efficiency of stripping of anti-VEGFR2 antibodies from surface molecules of VEGFR2 that are present not only on flat areas of the cell surface but also from inside of the pits that remain blocked at the plasma membrane. Scale bars are shown as insets at the bottom right side of each image.
